# Metabolic regulation of quiescence in plants

**DOI:** 10.1111/tpj.16216

**Published:** 2023-04-13

**Authors:** Michael J. Considine, Christine H. Foyer

**Affiliations:** ^1^ The UWA Institute of Agriculture and the School of Molecular Sciences The University of Western Australia Perth Western Australia 6009 Australia; ^2^ The Department of Primary Industries and Regional Development Perth Western Australia 6000 Australia; ^3^ School of Biosciences, College of Life and Environmental Sciences University of Birmingham Edgbaston B15 2TT UK

**Keywords:** metabolism, T6P, TOR kinase, SnRK1, sugar signalling, ROS, ROP2, quiescence, dormancy, meristem

## Abstract

Quiescence is a crucial survival attribute in which cell division is repressed in a reversible manner. Although quiescence has long been viewed as an inactive state, recent studies have shown that it is an actively monitored process that is influenced by environmental stimuli. Here, we provide a perspective of the quiescent state and discuss how this process is tuned by energy, nutrient and oxygen status, and the pathways that sense and transmit these signals. We not only highlight the governance of canonical regulators and signalling mechanisms that respond to changes in nutrient and energy status, but also consider the central significance of mitochondrial functions and cues as key regulators of nuclear gene expression. Furthermore, we discuss how reactive oxygen species and the associated redox processes, which are intrinsically linked to energy carbohydrate metabolism, also play a key role in the orchestration of quiescence.

## Significance Statement

The biology of plant quiescence, which makes cells highly resistant to environmental stress, is complex and poorly understood. Recent developments in dissecting the reciprocal influences of cellular energy and redox metabolism and signalling with nuclear controls provide new insights into how this essential process is established and the developmental functions it serves.

## INTRODUCTION

Quiescence is a fundamental feature of plant life that enables developmental plasticity, accommodating seasonal and incidental changes in environment and nutrition. In this way quiescence, as defined further in Box 1, plays important roles in ecology and agriculture, in preconditioning seed banks to the environmental cues that signal impending favourable conditions or preventing cereal seed from precocious germination. In an evolutionary sense, the evidence strongly suggests that quiescence is essential for the fidelity of the somatic cell line (Cruz‐Ramírez et al., 2013; Heyman et al., 2014).

Box 1 **Delineating quiescence and dormancy**

Quiescence (noun) is a labile state of a cell, embryonic or meristematic structure, where division of the cell or cell population is repressed by non‐cell‐autonomous processes. Cell division may not have ceased or stalled, and evidence suggests that mitosis continues even in stem cell populations. The horticulture and arboriculture community commonly refer to para‐ or eco‐dormancy, and the branching and shade avoidance communities may refer to bud dormancy. These latter modes are included within the definition of quiescence.

Dormancy (noun) refers to a quiescent embryonic or meristematic structure that is latent, not labile, whereby the mode of quiescence is entrained developmentally, commonly by seasonality. This definition only applies at a cellular level in mammalian and unicellular organisms, for example by the prevalence of particular protein regulatory modules, notably DREAM, and where this state is known as G0. There is no conclusive evidence of G0 in multicellular plants. The state of dormancy in seeds and perennating buds may be autonomous and defined by physical isolation, such as occlusion of plasmodesmata.

At a basic level, quiescence is a property of the cell cycle. Regulators of the eukaryotic cell cycle are widely conserved (Harashima et al., 2013). However, the coupling between the cell cycle and quiescence is less clear in plants than in other organisms (Velappan et al., 2017). In single‐cell organisms, the cell cycle is tightly linked to metabolism, in a way that temporally separates DNA synthesis from the potential mutagens of reactive oxygen species (ROS) that derive from the respiratory electron transfer chain in mitochondria (Coller, 2019; Salazar‐Roa & Malumbres, 2017). The energy requirements for DNA synthesis during the G1/S phase are met by glycolysis, whereas mitochondrial respiration is activated at the G2/M phase. The availability of oxygen and adenylates serve as metabolic checkpoints of the cell cycle, whereas genotoxic stress triggers a reprogramming of mitochondrial metabolism to ensure energy sufficiency during quiescence. Hence, there is a bi‐directional regulatory relationship between the cell cycle and metabolism (Kaplon et al., 2015).

Starvation is a primary driver of quiescence, and in single‐cell organisms, nutrient limitation and quiescence are the predominate state of being (Dworkin & Harwood, 2022; Soontorngun, 2017; Zhang & Cao, 2017). In mammalian cells, quiescence is associated with a considerable decline in basal metabolic rate, ATP levels, energy requirements and biosynthesis, most notably protein synthesis (Marescal & Cheeseman, 2020). There is recent evidence that the role and number of mitochondria declines during quiescence, facilitating a shift towards non‐fermentative aerobic respiration (Hocaoglu & Sieber, 2023), and the role of mitochondrial retrograde signalling is also discussed herein.

The relationship between metabolism and quiescence in higher plants is shaped by context. The context of the physiology and the developmental state of a plant or organ has a considerable role to play (Sablowski & Gutierrez, 2022; Velappan et al., 2017). We address physiological and developmental contexts throughout this review. In particular, we highlight the role of quiescence in meristematic tissues, which invariably act as a sink, and hence modulation by light will not be considered in depth (Fernández‐Milmanda & Ballaré, 2021; Schneider et al., 2019; Wang et al., 2020).

Clarity of language is important. Quiescence is a primary feature of cell division, meristem formation, stem cell regulation, cell differentiation, algal populations, seed dormancy and germination, root and shoot branching, perennial plant phenology and stress biology. Each of these fields have specific terminologies or have adopted slightly different meanings for particular terms. The term dormancy is one that has historically been confounding in plants (Baskin & Baskin, 2004; Considine & Considine, 2016; Velappan et al., 2017). Box 1 defines the terms quiescence and dormancy, as considered in this review. The discussion here largely attends higher plants, however, with limited attention to dormancy *sensu stricto*, where the context of life history prevails and where considerable knowledge gaps remain. We focus on reversible quiescence and exclude terminal differentiation and senescence. We also consider the roles of starvation and hypoxia as tools to understand developmental modes of quiescence.

For the purposes of this discussion, we constrain the definition of metabolism to mean primary metabolism, whereas for metabolic regulation, we adopt the approach of Miyazawa and Aulehla (2018) in separating metabolic regulation into bioenergetic functions and signalling. This includes signalling modes of feedback processes, plus redox and post‐translational modifications (PTMs). The roles of transcription factors (Luo et al., 2021; Zhang et al., 2022), such as those of the TCP (TEOSINTE BRANCHED 1/CYCLOIDEA/PROLIFERATING CELL FACTOR) and ERF (ethylene response factor) families, and hormones (García‐Gómez et al., 2021; Shimotohno et al., 2021) are integrated into the discussion where interaction with primary metabolism is known or postulated, but for a more detailed review we refer the readers to recent literature. In framing this review, we also acknowledge recent contributions on shoot branching (Barbier et al., 2019), the signalling roles of sugars (Wingler & Henriques, 2022), nutrients (L. Li et al., 2021), SNF1‐related protein kinase (SnRK1) and trehalose‐6‐phosphate (T6P) (Baena‐González & Lunn, 2020; Crepin & Rolland, 2019; Fichtner & Lunn, 2021), and the TARGET OF RAPAMYCIN (TOR) kinase (Artins & Caldana, 2022; Meng et al., 2022). We also point to notable recent reviews of cell cycle processes within plants (Desvoyes et al., 2021; Gutierrez, 2022; Sablowski & Gutierrez, 2022) and beyond (Huber et al., 2021; Ludikhuize & Rodríguez Colman, 2021; Marescal & Cheeseman, 2020).

## CELLULAR MECHANISMS TO SENSE AND ACCLIMATE TO ENERGY AVAILABILITY

Changes in energy status can influence plant growth or quiescence by metabolic sensing through enzymes that directly bind with sugars, particularly invertase (INV) and hexokinase (HXK), as well through influencing the activity of the protein kinase complex TOR kinase (reviewed by Artins & Caldana, 2022; Baena‐González & Lunn, 2020; Crepin & Rolland, 2019; G. Li et al., 2021; Meng et al., 2022; Wingler & Henriques, 2022). All of these enzymes are either directly regulated by redox post‐PTMs or are indirectly modulated by changes in cellular redox state (Heneberg, 2019; Sarbassov & Sabatini, 2005; Wurzinger et al., 2017). T6P has also emerged as an important cellular cue (Fichtner & Lunn, 2021), whereas pyruvate dehydrogenase kinase (PDK) has been implicated in metabolic quiescence in mammalian stem cells and may integrate with the tuning of quiescence via mitochondrial activity (Olson et al., 2016; Takubo et al., 2013). In the following discussion, we place emphasis on modes of energy‐related signalling that have been enlightened by recent studies, particularly in the past 5 years.

### The SnRK1‐bZIP module

**Figure 1:**
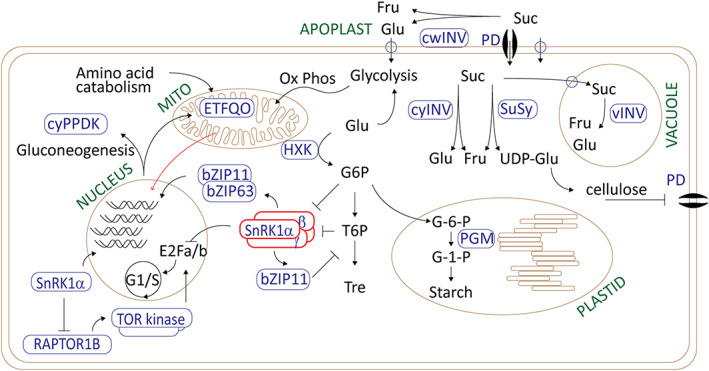
Metabolic regulation of plant meristematic quiescence by the SNF1‐related protein kinase (SnRK1), integrating changes in sugar metabolism. In conditions of local metabolic depletion, several functions of the sugar metabolic pathways are regulated in a manner that de‐represses SnRK1. The SnRK1a subunit retains a default function, where it is able to translocate to the nucleus to transcriptionally regulate catabolic functions, notably leading to gluconeogenesis as well as to amino acid catabolism via the reprogramming of mitochondrial metabolism. Mitochondrial retrograde signalling (red arrow) facilitates nuclear reprogramming and SnRK1 function (Figure 2). Sucrose (Suc) levels are restricted by the activity of sucrose synthase (SuSy), which is positively regulated by SnRK1 (not depicted). SuSy activity supports cellulose synthesis in the presence of UDP‐glucose (UDP‐Glu), which may reinforce plasmodesmatal (PD) occlusion, a common feature of dormancy transitions in the latent buds and cambium of perennial plants. Alternatively, in the presence of ADP‐glucose, SuSy activity may support starch synthesis. In this way, glucose‐6‐phosphate (G6P) and trehalose‐6‐phosphate (T6P) levels are restricted, reinforcing or enabling the activation of SnRK1. SnRK1 can function as a transcriptional regulator or through phosphorylation, notably of bZIP transcription factors. At least two of SnRK1 phosphorylation targets restrict cell division (G1/S): the E2Fa/b transcription factors, and the RAPTOR1B subunit of the TOR kinase, which is a positive regulator of the E2Fa/b. The activities of other metabolic enzymes, such as invertase (INV) and hexokinase (HXK) function to antagonize SnRK1 and support cell division. Abbreviations: cwINV, cell wall invertase; cyINV, cytosolic invertase; cyPPDK, cytosolic pyruvate, phosphate dikinase; ETFQO, electron transfer flavoprotein:ubiquinone oxidoreductase; Fru, fructose; Glu, glucose; Ox Phos, oxidative phosphorylation; PGM, phosphoglucomutase; Tre, trehalase; vINV, vacuolar invertase.

The SnRK1 module is considered to be the counterpart master regulator of cellular quiescence to the TOR kinase (Margalha et al., 2019). Whereas SnRK1 functions in conditions of low energy or stress response, activating catabolic processes, TOR kinase functions under energy‐replete conditions, activating anabolic metabolism and cell division. Interestingly, a recent study indicated the potential of the SnRK1α subunit to act independently of other subunits and in a default manner (Ramon et al., 2019). Moreover, the BASIC LEUCINE ZIPPER (bZIP) transcription factors have been strongly implicated in functioning downstream of SnRK1 in developmental adjustment and response to a transient energy crisis, as illustrated in Figure 1.

Although SnRK1 shares many conserved functions of 5′‐AMP‐activated protein kinase (AMPK) and SUCROSE NON‐FERMENTING 1 (SNF1), it is not influenced by adenine nucleotide charge, notably AMP, which is a central feature of metabolic regulation in its mammalian and yeast counterparts (Emanuelle et al., 2015). SnRK1 is however negatively regulated by sugar phosphates, including T6P (Baena‐González & Lunn, 2020; Fichtner & Lunn, 2021), as discussed below. Further, the catalytic α subunit is able to act independently of the β and γ regulatory subunits, translocating to the nucleus to promote the expression of catabolic functions such as DARK INDUCED 6 (DIN6)/ASPARAGINE SYNTHASE (Ramon et al., 2019). The subcellular localization of SnRK1α appears to be a key determinant of the outcome of its function. The myristoylated SnRK1β subunits can restrict the nuclear localization of SnRK1α and inhibit target gene induction, influencing leaf and root development (Ramon et al., 2019). Meanwhile, the cytosolic localization of SnRK1α in the presence of abscisic acid (ABA) appears to inhibit the TOR kinase (Belda‐Palazón et al., 2022). The cytosolic RAPTOR1B subunit of the TOR kinase is phosphorylated by SnRK1α (Nukarinen et al., 2016). Hence, the activation of SnRK1 may have different outcomes in different subcellular locations.

SnRK1 directly regulates the E2Fa and E2Fb transcription factors, which are targets of the TOR kinase that controls the expression of S‐phase genes. Moreover, SnRK1 directly phosphorylates E2Fa/b under the energy‐depleted conditions of darkness and hypoxia, regulating its proteolytic degradation and G1 cell cycle arrest (Son et al., 2022). Hence, E2Fa/b is a mutual target of the TOR kinase and SnRK1, acting in direct antagonism. Whether this function requires ABA, is unique to dark or hypoxic conditions or indeed the full SnRK1 complex, remains to be explored.

The bZIP transcription factors are important SnRK1‐dependent vectors of quiescent responses. The C‐type bZIP63 is phosphorylated *in vivo* by SnRK1, resulting in dimerization with other S‐ or C‐type bZIPs and enabling the transcriptional activation of genes involved in catabolism (Mair et al., 2015). Furthermore, the S‐type bZIPs together with bZIP63 directly regulate the expression of the mitochondrial electron‐transfer flavoprotein:ubiquinone oxidoreductase (ETFQO), which plays a critical role in dark‐induced starvation as well as other genes encoding enzymes in amino acid catabolism and gluconeogenesis, notably the cytosolic pyruvate phosphate dikinase (cyPPDK) (Henninger et al., 2022; Pedrotti et al., 2018). The role of bZIPs, particularly bZIP11 and bZIP63, in mediating SnRK1‐dependent functions is well established: for example, in the responses of root system architecture to dark‐induced starvation (Muralidhara, 2022; Weiste et al., 2017) and in the metabolic process of seed maturation (Alonso et al., 2009). This is a critical stage in the onset of quiescence that prepares the tissues for dormancy. bZIP11 also regulates trehalase activity, promoting the degradation of T6P, and restricts gene expression of T6P synthase (Ma et al., 2011; S. Zhang et al., 2021).

The SnRK1α catalytic subunit (KIN10) is redox regulated, favouring activation in the reducing environment of dividing cells (Wurzinger et al., 2017). The phosphorylation of targets including bZIP63 requires the reduction of Cys200 of KIN10. Curiously, such regulation would repress SnRK1 functions in the stem cell niche, which is in an oxidized and hypoxic state (Considine et al., 2017; Considine & Foyer, 2021; Shukla et al., 2019; Weits et al., 2019). It has yet to be determined whether this process functions when the SnRK1 complex is formed or, indeed, *in vivo*.

The overexpression of the SnRK1α subunit in fruit or tubers leads to the expression of sucrolytic enzymes, notably sucrose synthase (SuSy), which maintains an equilibrium between sucrose and hexose substrates, and promotes the accumulation of cellulose or starch (Luo et al., 2020; Ren et al., 2019). This is consistent with the decline in T6P by regulation of trehalase (S. Zhang et al., 2021). The cells of fruit and tubers have entered the endocycle, which may establish a distinct context for the function and regulation of SnRK1 compared with the canonical cell cycle. Nevertheless, there is considerable evidence for SnRK1 activation and the transcriptional regulation of bZIP transcription factors in establishing catabolism and quiescence in fruit. Key remaining unknowns are addressed in Box 2.

Box 2 **Open questions**

1. Much of our knowledge of the function of SnRK1 is restricted to dark‐ and hypoxia‐induced starvation, including recent evidence for a default activity of SnRK1α. Can the SnRK1 function be dissected from these conditions, which have unique signalling consequences?
2. Evidence suggests that the plant stem cells are oxidized and hypoxic and the metabolic maintenance of this state preserves the fidelity of the germline; however, SnRK1, a pivotal agent of quiescence, is repressed in oxidized conditions. How can these insights be reconciled?
3. How does metabolism, particularly organelle metabolism, influence the subcellular localization and function of SnRK1 and TOR kinase?
4. What role does the regulation of cell–cell movement via plasmodesmata play in shaping the metabolic regulation of quiescence?

### The ROP2‐TOR module

The TOR kinase has a central role in nutrient and energy signalling (Artins & Caldana, 2022; Meng et al., 2022). It integrates cellular cues, including hormone and nutritional status, to activate the cell cycle, particularly through the phosphorylation of the E2Fa and E2Fb transcription factors. Below we focus on the upstream metabolic regulators of the TOR kinase, particularly the Rho‐related protein from plants 2 (ROP2).

ROP2 is an important upstream regulator of TOR kinase functions in cell growth. The negative regulation of ROP2 is associated with quiescence. ROP2 is a member of a small family of plant‐specific Roh‐like GTPases that play critical roles in cell polarity (Feiguelman et al., 2018). ROP2 is a small GTPase that is regulated by the binding of GTP (active) or GDP, which is dependent on the activity of ROP‐GEF (GDP/GTP exchange factors), ROP‐GAP (GTPase activating proteins) and other interacting proteins. These proteins in turn appear to be regulated differently depending on the cellular context. ROP2 is typically associated with the plasma membrane. When activated by GTP, ROP2 binds directly with the TOR kinase, leading to its activation (Schepetilnikov et al., 2017).

Constitutively active (*CA‐rop2*) or dominant negative (*DN‐rop2*) mutants of ROP2, which permanently bind GTP or GDP, respectively, display opposite developmental phenotypes. The Arabidopsis *DN‐rop2* seeds display greater seed dormancy and are hypersensitive to ABA, a well‐known hormonal regulator of dormancy (Li et al., 2001). The *CA‐rop2* seedlings show a constitutive photomorphogenesis phenotype, whereas *DN‐rop2* have longer hypocotyls in the dark, a phenomenon that is dependent on the downstream regulation of the TOR kinase (Li et al., 2017). The *DN‐rop2* shoots show a marked loss of apical dominance, resulting in greater outgrowth of axillary buds (Li et al., 2001).

ROP2, which is regulated by glucose, inorganic nitrogen and amino acids in roots and shoots, is an upstream regulator of the TOR kinase (Li et al., 2017; Liu et al., 2021; Tulin et al., 2021). Hormones such as auxin and brassinosteroids influence ROP2 functions (Li et al., 2001), whereas ABA signalling is negatively regulated by ROP2 (Hwang et al., 2011), which may explain the ABA‐hypersensitivity of *DN‐rop2* seeds (Li et al., 2001). The receptor‐like kinase FERONIA is another important upstream regulator of ROP2 (Duan et al., 2010). FERONIA transduces cell mobile signals via rapid alkalinization factor (RALF) peptides that interact with ROP‐GEF to activate ROPs, including ROP2. Mutants of *FER* showed root‐hair defects as well as stunted shoot growth. The RALF1‐FER complex phosphorylates eIF4E1, an early translation initiation factor, promoting the translation of ROP2 (Zhu et al., 2020).

ROP2 binds to HYPOXIA‐INDUCIBLE UNIVERSAL STRESS PROTEIN 1 (HRU1) and RESPIRATORY BURST OXIDASE HOMOLOG PROTEIN D (RBOHD) during anoxia/hypoxia (Gonzali et al., 2015), enabling an oxidative burst that triggers local and systemic signalling, including the transcriptional upregulation of *ROP‐GAP* to enable feedback (Baxter‐Burrell et al., 2002). Thus, oxygen and energy, together with hormonal cues, converge on ROP2 to modulate TOR kinase and in turn influence cell division and growth (Considine, 2017). The metabolic regulation of ROP‐GEF and the role of other ROP‐family members in metabolic regulation remains largely unexplored.

The mechanism of the loss of apical dominance in *DN‐rop2* invites some consideration. Apical dominance is primarily governed by the hormones auxin, cytokinin, strigolactone and ABA, together with sucrose (Barbier et al., 2019). To date, the role that oxygen, through ROS, redox regulation and hypoxia signalling, plays in apical dominance remains unclear. Quiescent axillary buds are oxidized (Chen et al., 2016; Porcher et al., 2020, 2021) and presumed to be hypoxic. Hence, the inability to activate ROP2 with GTP may alter ROS metabolism. GTP‐ROP2 is also required to activate alcohol dehydrogenase, which enables survival following waterlogging (Sun et al., 2018). The branching phenotype of *DN‐rop2* may indicate the integration of ROS and sugar availability signals in the regulation of metabolic and cell cycle quiescence.

The stability of the TOR complex is required for the orderly regulation of the plasmodesmatal aperture and source‐to‐sink transport (Brunkard et al., 2020). Here, the loss of a Reptin protein, believed to function in stabilizing the TOR complex, or indeed the loss of the LST8 protein component of the TOR complex, leads to increased plasmodesmatal aperture. The orchestrated movement via plasmodesmata is essential for the establishment and maintenance of stem cell identity (Kitagawa & Jackson, 2017). In mature leaves, the loss of LST8 or chemical inhibition of the TOR kinase increased the movement of ectopic GFP via the phloem. Moreover, a second plasmodesmata mutant identified in this forward‐genetic screen mapped to a gene encoding a mitochondrial SEL1‐like repeat containing (SLR) protein, which cofractionates with the mitochondrial ATP synthase (Brunkard et al., 2020). Both mutants of the SLR and Reptin exhibited the low TOR kinase activity and gene expression profiles characteristic of quiescence and catabolism. The inhibition of glycolysis or mitochondrial respiration increases plasmodesmatal aperture, indicating that the regulation of plasmodesmata by the TOR kinase is a function of disruption of respiration. Consistent with this conclusion, the disruption of mitochondrial or chloroplast ROS metabolism also alters plasmodesmatal aperture and cell functions (Benitez‐Alfonso et al., 2011; Burch‐Smith et al., 2011; Burch‐Smith & Zambryski, 2010; Stonebloom et al., 2012). The establishment and function of plasmodesmata is complex (Roeder et al., 2022), and it is certainly important for meristem identity and fate, including quiescence. The functioning of plasmodesmata plays a key role in the seasonal control of dormancy transitions in trees (Miskolczi et al., 2019; Tylewicz et al., 2018). Understanding the metabolic regulation of this process remains an open question (Box 2).

## CARBOHYDRATE METABOLISM AND SIGNALLING

### Starch metabolism

The role of starch in growth, more broadly, was recently reviewed (Smith & Zeeman, 2020). The influence of starch accumulation on the decision to grow or quiesce is evident in the environmental sensitivity of mutants deficient in starch metabolic enzymes. For example, the growth of mutants lacking the plastid phosphoglucomutase (PGM) are highly sensitive to the transitory depletion of starch under short‐day conditions. Mutants deficient in cytosolic PGM show dwarf phenotypes, which is amplified by the depletion of the plastid isoform (Malinova et al., 2014).

Hypoxia, which is discussed further in the sections below, triggers quiescence. Survival following a period of hypoxia requires starch accumulation (Loreti et al., 2017). Hypoxia is lethal to mutants lacking PGM, and this is partially dependent on sucrose levels. Remarkably, the response of core anaerobic genes is repressed by starvation; many of these genes serve to generate energy intermediates, for example alcohol dehydrogenase (Loreti et al., 2017).

The latent axillary buds of herbaceous annuals and woody perennials express conserved starvation‐induced genes (Tarancón et al., 2017). Interestingly, then, the *pgm* knockdown lines of hybrid Aspen showed very little growth phenotype under benign conditions (Wang et al., 2022). Although the lines were deficient in starch, seasonal responses in bud quiescence and dormancy were unaffected.

### Sucrose and trehalose signalling and metabolism

Sink tissues rely on sucrose as an energy source, as well as the breakdown of glucose from storage metabolites. Sucrose is unloaded from the phloem via symplastic or apoplastic pathways, leading to points of metabolic control (Figure 1; Ruan, 2014). Here we give a brief review and update of recent additions to the literature. We focus primarily on sucrose metabolism rather than translocation, although the role of translocation in source/sink relationships, and thus the decision of where to direct growth, is also influenced at this level (Liu et al., 2019; Otori et al., 2019; Zakhartsev et al., 2016).

Sucrose participates in hormone‐like functions that interact with other cues to govern apical dominance and bud outgrowth (F.F. Barbier et al., 2015, 2019). Sucrose functions as a mobile signal in triggering bud outgrowth following decapitation (Mason et al., 2014). Even in the absence of decapitation, feeding ectopic sucrose to the bud was sufficient to promote growth and repress the expression of *BRANCHED 1* (*BRC1*), a negative regulator of bud outgrowth.

The metabolic function of sucrose is indicated in studies showing the influence of leaf area on bud outgrowth. For example, the reduction in leaf area by defoliation led to rapid changes in axillary bud outgrowth of sorghum, together with the expression of marker genes for quiescence, the cell cycle and starvation, regardless of the proximity of the leaf to the bud (Kebrom & Mullet, 2015). Nevertheless, non‐metabolizable analogues of sucrose can also trigger outgrowth (F. Barbier et al., 2015), suggesting that disaccharide signalling explains at least part of the effect on outgrowth. Signalling may be mediated by T6P or by enzymes that directly interact with sucrose, notably INV and SuSy.

When unloaded via the apoplastic pathway, sucrose is hydrolysed by cell wall invertase (cwINV) to hexoses, which are then transported across the membrane. Following earlier studies illustrating its role in maintaining source/sink balance (reviewed by Ruan, 2014), the cwINV was recently shown to be critical for ovule initiation, in a manner independent of carbon availability, indicating a signalling mode (Liao et al., 2020). Alternatively, a comparison of two species of bulb (*Lycoris* spp.), differing in regeneration rates, showed a clear discrimination between sucrose metabolic functions (Ren et al., 2022). The prolific species (*Lycoris sprengeri*) preferentially unloaded sucrose via cwINV at an important early stage of propagation, indicating a clearly important metabolic function in determining growth.

The role of vacuolar invertase (vINV) as a signalling enzyme has recently been established. Feeding sucrose to excised, etiolated *Solanum tuberosum* (potato) shoots, promotes branching in a manner that depends on the activity of vINV and cytokinin signalling (Salam et al., 2017, 2021). The hydrolysis of sucrose is impeded in *vinv* knockdown mutants, resulting in elevated sucrose and depleted hexose levels, particularly following cold storage. In this system, intact tubers of *vinv* lines show elevated branching compared with the wild type (WT; Salam et al., 2017). In *in vitro* single‐node, etiolated stems, *vinv* mutants show a weakened sensitivity to sucrose compared with the WT (Salam et al., 2021). These data suggest that vINV activity, rather than sucrose or hexose levels, is important for branch activity in potatoes. However, whether catalytic activity is required for this function remains unknown. A more structural metabolic function of the cytosolic invertase (cyINV) was recently shown (Barnes & Anderson, 2018). Mutants deficient in cyINV activity have reduced anisotropic growth and abnormal cellulose organization, indicating a major function for this enzyme in supplying UDP‐glucose for cellulose synthesis.

Although SuSy contributes to sink strength (Ruan, 2014), this enzyme does not contribute to leaf starch synthesis (Fünfgeld et al., 2022). The mechanistic differences between source and sink tissues, such as the seed endosperm, where elevated SuSy activity increases starch and ADP‐glucose levels (Li et al., 2013), remain to be elucidated. SuSy is also discussed in the section on SnRK1‐bZIP.

The levels of T6P, which is a homeostatic sensor of sucrose and energy status, closely follow sucrose under a range of conditions (Yadav et al., 2014), although at three or four orders of magnitude lower concentrations. The function of T6P in regulating the decision to grow or quiesce has been recently reviewed (Baena‐González & Lunn, 2020; Fichtner & Lunn, 2021). The remarkable homeostasis between sucrose and T6P was best illustrated when bacterial (*Escherichia coli*) trehalose metabolic enzymes were introduced into Arabidopsis (Yadav et al., 2014). The heterologous expression disrupted the native plasticity of T6P levels to altered conditions, resulting in a shifted T6P:sucrose homeostasis and distinct phenotypes of the mutant lines. Nevertheless, the homeostasis remained resilient within lines under variable conditions. Following earlier observations that T6P was concordant with the influence of sucrose on bud outgrowth (Fichtner et al., 2017), Fichtner et al. (2021) used a similar heterologous system to that of Yadav et al. (2014) to manipulate T6P levels specifically within the axillary bud or vascular tissues. Altered synthesis or degradation of T6P locally resulted in altered rates of bud outgrowth, such that elevated bud‐specific T6P:sucrose accelerated bud outgrowth, whereas lower levels resulted in a considerable delay to bud outgrowth kinetics. These insights show that the T6P metabolite is the relevant cue, rather than the catalytic activity of the enzymes acting on T6P (Fichtner et al., 2021). The influence of T6P on the metabolic activity of the sink has also been established in seeds, where elevated T6P promotes starch accumulation and growth of the embryo (Meitzel et al., 2021). Although these data collectively indicate the importance of metabolite levels of T6P rather than the activity of corresponding enzymes, functional analysis of the T6P synthase (TPS) in Arabidopsis shows that non‐catalytic domains of the enzyme are important for the correct localization and orderly activation of TPS, leading to T6P synthesis (Fichtner et al., 2020).

The mechanisms of T6P functions include the ability to inhibit catalytic activity of SuSy (Fedosejevs et al., 2018), resulting in feedback inhibition and contributing to T6P:sucrose homeostasis. In addition, T6P directly inhibits the SnRK1 of Arabidopsis (Baena‐González & Lunn, 2020; Zhang et al., 2009). Interestingly, this intermediate is absent from mature or senescing tissues, which may explain the transitory delay to bud outgrowth in Arabidopsis mutants with reduced T6P:sucrose ratio (Fichtner et al., 2021), such that the intermediate is developmentally transient.

### Glucose signalling and metabolism

Glucose signalling pathways are typically considered to be HXK dependent or independent. The HXK‐independent pathway, or glycolytic pathway, largely comprises modes that have been discussed above, notably the TOR kinase. Here, we briefly introduce HXK‐dependent signalling (reviewed by Aguilera‐Alvarado & Sanchez‐Nieto, 2017). The involvement of HXK in glucose signalling was classically illustrated by studies showing that catalytically inactive HXK1 of Arabidopsis was able to restore glucose‐dependent developmental phenotypes to *gin2‐1* mutants, which are defective in the *HXK1* gene (Moore et al., 2003). The catalytic activity of HXK1 is not required for branching in Arabidopsis. Mutants lacking HXK1 (*gin2*) have delayed branching and a reduced number of branches at bolting, a phenotype that is largely restored by the expression of a catalytically inactive HXK1 (Barbier et al., 2021). Consistent with this finding, the stimulatory effect of sucrose on bud outgrowth was suppressed by a competitive inhibitor of HXK in rose. Mannose, which is phosphorylated by HXK but subsequently metabolized only weakly, was also able to stimulate bud outgrowth, whereas 3‐*O*‐methylglucose, which is neither phosphorylated nor metabolized further, did not (Barbier et al., 2021).

Although the HXK1 protein is localized to the outer mitochondrial membrane, it is also found in the nucleus, where it binds to target genes and can even phosphorylate transcription factors (L. Li et al., 2021). It can therefore be considered as a moonlighting protein (Aguilera‐Alvarado & Sanchez‐Nieto, 2017; Rodríguez‐Saavedra et al., 2021). Certain mammalian HXKs are also anchored to the outer mitochondrial membrane and are shown to interact with voltage‐dependent anion channels, an abundant mitochondrial porin, as well as perform a number of other functions that are potentially relevant to energy metabolism but are independent of glucose phosphorylation, such as, the regulation of mitochondrial HXK by reactive oxygen and nitrogen species (Heneberg, 2019). Such regulation has been characterized substantially in mammals but remains to be explored in plants.

## MITOCHONDRIA

**Figure 2:**
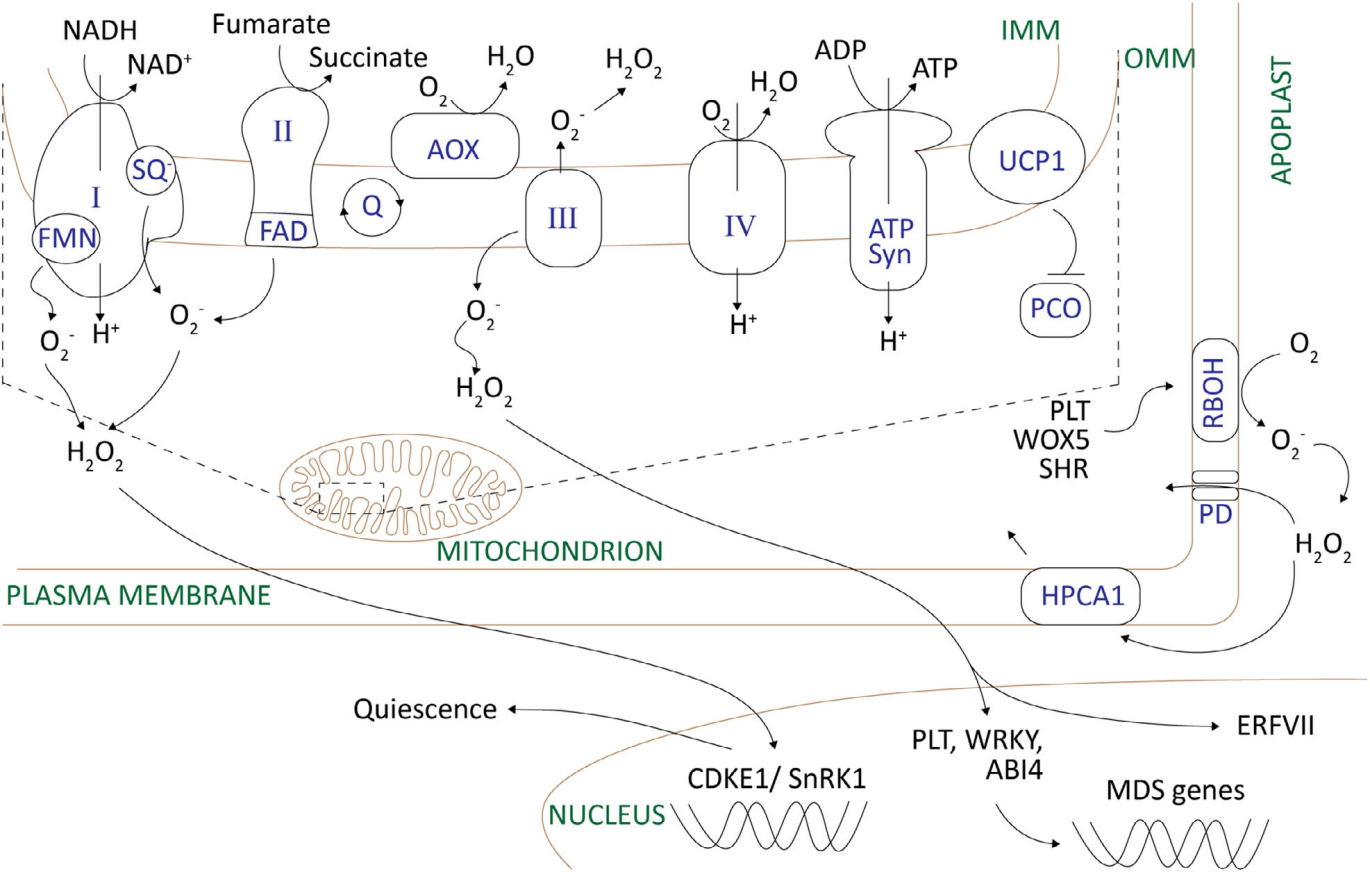
Schematic representation of the central role of mitochondria in oxygen and reactive oxygen species (ROS) signalling in the regulation of quiescence. Limitations in oxygen availability in the stem cell niche favour ROS production and oxidation, altering the regulation of plasmodesmata by TOR kinase and activating the expression of MITOCHONDRIAL DYSFUNCTION STIMULON (MDS) genes such as alternative oxidase (AOX), the function of which is to restrict metabolic ROS production while still maintaining cytoplasmic oxidation and quiescence. Like AOX, the mitochondrial UNCOUPLING PROTEIN 1 (UCP1) has been linked to mitochondria–nucleus retrograde signalling. In the case of UCP1, signalling leads to an inhibition of the cytoplasmic PLANT CYSTEINE OXIDASE (PCO) branch of the PROTEOLYSIS 6 (PRT6) N‐degron pathway. The CDKE1 member of the mediator complex is signalled via mitochondria to reinforce the cell cycle quiescence outcomes of SnRK1. Several transcription factors, PLETHORA (PLT), SHORT‐ROOT (SHR) and ethylene response factor (ERF115), maintain stem cell quiescence and activate apoplastic ROS production by the membrane‐bound NADPH oxidases (RBOH) in order to create an oxidative micro‐environment that allows asymmetric periclinal cell divisions. The compartment‐specific regulation of ROS (H_2_O_2_, O_2_^−^) production therefore plays a key role in the regulation of quiescence. Abbreviations: I, complex I; II, complex II; III, complex III; IV, complex IV; ABI4, ABSCISIC ACID‐INSENSITIVE 4; ATP Syn, ATP synthase/complex V; FAD, flavin adenine dinucleotide; FMN, flavin mononucleotide; IMM, inner mitochondrial membrane; NAD(H), nicotinamide adenine dinucleotide (reduced); OMM, outer mitochondrial membrane; Q, ubiquinone; SQ^−^, semiquinone radical; WOX5, WUSCHEL‐RELATED HOMEOBOX5.

Oxygen (O_2_) delivery is an effective prerequisite for cell functions because of its role in aerobic respiration that generates energy in the form of ATP through oxidative phosphorylation and the tricarboxylic acid (TCA) cycle (Figure 2). Mitochondria are by far the main consumers of oxygen in the cell (Considine & Foyer, 2021; Noctor & Foyer, 2016). Therefore, it is not surprising that oxygen deficiency changes the metabolism, signalling and protein composition of mitochondria. Oxygen deficiency impacts directly on mitochondrial energy metabolism, as cells are forced to use anaerobic respiration and reductive carboxylation, which favours enhanced ROS production (Shingaki‐Wells et al., 2014; Wagner et al., 2018). Hypoxia was found to alter mitochondrial morphology, composition and mass in mice nucleus pulposus cells through a HYPOXIA‐INDUCIBLE FACTOR 1α (HIF1α)‐dependent pathway (Madhu et al., 2020). In contrast, the mitochondria in the hypoxic and highly oxidizing environment of the root quiescent centre (QC) in *Zea mays* (maize) had much lower activities of TCA cycle enzymes but were otherwise structurally comparable with mitochondria in the adjacent tissues (Jiang et al., 2006). Remarkably, in the resurrection species *Haberlea*, the mitochondria of desiccated leaves retain a fully functioning respiratory chain, which resumes activity immediately upon hydration, whereas photosynthesis and plastid functions are delayed (Ivanova et al., 2022).

Mitochondria sense oxygen levels and initiate cellular responses to hypoxia, including the regulation of chaperones and heat‐shock proteins and transporters, as well as the expression of the ALTERNATIVE OXIDASE 1a (AOX1a) and other components of the alternative respiratory chain, such as NAD(P)H DEHYDROGENASE B2 (NDB2; Wagner et al., 2018). Signalling proteins that mediate the response have been identified, called the ‘MITOCHONDRIAL DYSFUNCTION STIMULON’ (MDS; de Clercq et al., 2013). The promotors of these genes are targeted by NO APICAL MERISTEM/ARABIDOPSIS TRANSCRIPTION ACTIVATION FACTOR/CUP‐SHAPED COTYLEDON (NAC) transcription factors (de Clercq et al., 2013), particularly ANAC013 and ANAC017/REGULATORS OF ALTERNATIVE OXIDASE 1a (RAO2). WRKY‐type transcription factors (WRKY15/WRKY40/WRKY63; van Aken et al., 2013; Vanderauwera et al., 2012) and ABSCISIC ACID INSENSITIVE 4 (ABI4; Giraud et al., 2009) are downstream regulators of mitochondrial retrograde signalling pathways. Like the WRKY transcription factors, which regulate a range of developmental programmes in response to environmental stresses (Rushton et al., 2010), ABI4 also regulates development, and is important in processes such as seed germination (Finkelstein et al., 1998), flower development (Shu et al., 2016) and lateral root formation (Shkolnik‐Inbar & Bar‐Zvi, 2010).

CYCLIN‐DEPENDENT KINASE E1 (CDKE1/RAO1) regulates AOX1a expression as part of the retrograde signalling hub from mitochondria as well as chloroplasts (Blanco et al., 2014; Ng et al., 2013). CDKE1 is predicted to be a subunit of the Arabidopsis mediator complex, which acts as a scaffold that bridges DNA‐bound transcription factors to RNA polymerase II, linking signalling input to transcription (Mathur et al., 2011). Nuclear‐localized CDKE1 interacts with the SnRK1α catalytic subunit KIN10, which integrates transcription networks of plant stress signalling, sugar metabolism and developmental programmes (Baena‐Gonzalez et al., 2007; Cho et al., 2012; Ng et al., 2013). Hence, there is clear evidence of a pivotal role for mitochondria in the metabolic regulation of quiescence, although much remains to be explored.

## ROS AND HYPOXIA

Developmental hypoxia is crucial to stem cell functions in animals and plants. Stem cells are housed in a hypoxic environment (Di Mattia et al., 2021) that both increases and limits mitochondrial ROS production and also increases the activation of mitochondrial systems that remove ROS (Guzy et al., 2005; Mailloux, 2020; Pucciariello & Perata, 2021; Shingaki‐Wells et al., 2014). Within this context, the concept that mammalian stem cells reside in a hypoxic micro‐environment that protects them from oxidative stress that would otherwise prevent self‐renewal and accelerate ageing, is widely accepted (G. Li et al., 2021; Mohyeldin et al., 2010). However, the physiological role of ROS in stem cells extends far beyond potential adverse effects. Intracellular ROS protection in mammalian stem cells participates in the orchestration of differentiation and cell fate, and they have a positive role on stem cell self‐renewal. ROS participate in the ‘waking up’ of the stem cells so that they can enter the cell cycle upon exit from quiescence (Lyublinskaya et al., 2015). A similar situation occurs in plant cells, such as those in the root apical meristem (RAM; de Simone et al., 2017).

ROS generation in plants is the driver and first requirement for many developmental processes, such as the cell cycle, pollen viability, microspore reprogramming towards sporophytic development, the regulation of female gametophyte patterning and the maintenance of embryo sac polarity, as well as the prevention of self‐pollination (de Simone et al., 2017; Sankaranarayanan et al., 2020; L. Zhang et al., 2021; Żur et al., 2021). It is not surprising therefore that mitochondrial ROS are suggested to be important signals in the regulation of MDS. The activation of anaerobic core genes by ETHYLENE RESPONSE FACTOR VII (ERF‐VII) transcription factors requires ROS signals (Sasidharan et al., 2021). A further mitochondrial retrograde signalling mechanism that links oxygen consumption to oxygen sensing involves UNCOUPLING PROTEIN 1 (UCP1) (Barreto et al., 2022). This inner mitochondrial membrane protein regulates nuclear gene expression by inhibiting the cytoplasmic PLANT CYSTEINE OXIDASE (PCO) branch of the PROTEOLYSIS 6 (PRT6) N‐degron pathway (Figure 2). These components integrate mitochondrial and nuclear functions during plant development as well as in response to low oxygen stress (Barreto et al., 2022).

The plasma membrane NADPH oxidases or respiratory burst oxidase homologues (RBOHs) that generate the superoxide anion (O_2_^−^) in the apoplast/cell wall space are the main source of ROS in cells subjected to developmental hypoxia (Considine & Foyer, 2014; Liu et al., 2017; Pucciariello & Perata, 2017). Superoxide is then converted into hydrogen peroxide (H_2_O_2_), either by spontaneous dismutation or by the action of the enzyme superoxide dismutase. Apoplastic ROS production under hypoxia is controlled at the transcriptional and post‐transcriptional levels. For example, the RELATED TO AP‐2.12 (RAP2.12) transcription factor regulates the expression of *HYPOXIA‐RESPONSIVE UNIVERSAL STRESS PROTEIN 1* (*HRU1*) as well as *RBOHD* under hypoxia (Gonzali et al., 2015). Interactions between HRU1 and RBOHD regulate anoxia‐induced hydrogen peroxide accumulation (Gonzali et al., 2015). In addition, HRU1 interacts with the hypoxia‐activated GTP‐ROP2, which is also required for ROS accumulation and survival under anoxia (Baxter‐Burrell et al., 2002), and is a direct regulator of the TOR kinase, as discussed above.

Waves of RBOH‐dependent ROS accumulation are important in cell‐to‐cell communication (Fichman et al., 2021, 2022; Zandalinas et al., 2019, 2020; Zandalinas, Fichman, et al., 2020). Auto‐propagating waves of ROS, calcium and electric signals function together to generate rapid systemic cell‐to‐cell communication (Wang et al., 2019), playing a pivotal role in local and systemic responses, leading to stress acclimation and survival (Fichman & Mittler, 2021a, 2021b; Waszczak et al., 2018; Zandalinas et al., 2020; Zandalinas, Fichman, et al., 2020).

Silencing genes coding for RBOH in *Solanum lycopersicum* (tomato) led to a loss of apical dominance and increased branching (Chen et al., 2016). High hydrogen peroxide levels are consistently correlated with the quiescence of axillary buds, whereas the chemical inhibition of the antioxidant glutathione represses bud outgrowth (Porcher et al., 2020, 2021). As mentioned earlier, the sucrose signal following decapitation, which triggers bud outgrowth, reportedly travels at 150 cm h^−1^ (Mason et al., 2014). Meanwhile systemic ROS wave signalling in response to touch or wounding travels several times faster (Miller et al., 2009). Electrical and Ca^2+^ signals, together with small peptides, also play prominent roles in systemic signalling (Johns et al., 2021); however, these remain to be explored in the context of apical dominance or bud outgrowth.

There are strong interactions between ROS and nitric oxide (NO) signalling in plant responses to hypoxia, during which the phytoglobin/NO pathway (Pgb/NO cycle) helps to maintain cellular redox and energy levels. The mitochondrial alternative oxidase (AOX) preserves the mitochondrial redox state by preventing the over‐reduction of the respiratory electron transport chain and minimizing the generation of mitochondrial NO and ROS. Under hypoxia, however, AOX activity can accelerate NO production, followed by NO conversion to nitrate by the enhanced expression of class‐1 phytoglobin (Pgb1; Zafari et al., 2022). The activity of AOX reduces electron transfer between complexes III and IV under normoxia, limiting NO accumulation. In contrast, under hypoxia, AOX serves to increase nitrite‐dependent NO production (Sasidharan et al., 2018; Vishwakarma et al., 2018). NO plays an important role in the post‐PTM of target proteins. It is proposed that *S*‐nitrosylation plays a mechanistic role in regulating the N‐degron pathway, which regulates gene expression under hypoxia, although the precise target remains unknown (Castillo et al., 2018; Giuntoli & Perata, 2018). NO‐mediated *S*‐nitrosylation also triggers the degradation of the key ABA‐signalling protein ABI5, enabling germination following quiescence (Albertos et al., 2015), while also being able to activate ethylene production by the *S*‐nitrosylation of biosynthetic enzymes (Li et al., 2016). The *S*‐nitrosoglutathione (GSNO) reductase, a master regulator of NO signalling, is itself modified by S‐nitrosylation under hypoxia, leading to its degradation by autophagy (Zhan et al., 2018). Further important targets under hypoxic conditions include mitochondrial proteins such as cytochrome *c* oxidase (COX) and aconitase, which are also regulated by *S*‐nitrosylation (Igamberdiev et al., 2014), and RBOHD, where *S‐*nitrosylation reduces its activity (Yun et al., 2011). Interactions between superoxide and NO in mitochondria initiate an ROS‐dependent NO degradation pathway that involves thioredoxins (Wulff et al., 2009). AOX appears to control ROS–NO interactions in mitochondria that control defensive responses under hypoxia.

RBOHD and calcium signalling are required for the ROS‐dependent expression of ERF transcription factors such as ERF6 and ERF73 (Sewelam et al., 2013). ERF6 plays an important role in the regulation of antioxidant gene expression, limiting the spread of ROS signals that modulate plant growth and defence in response to biotic and abiotic stresses (Sewelam et al., 2013). NO targets ERF‐VII for proteasomal degradation through an O_2_‐dependent N‐degron pathway (Hartman et al., 2019). Ethylene‐dependent signalling pathways are also essential for the stress‐induced expression of AOX and ERF‐VII genes (Ederli et al., 2006).

The PLETHORA (PLT) family of AP2‐type transcription factors are master regulators of root formation and development that are highly expressed in the QC and SCN (Aida et al., 2004; Galinha et al., 2007). The action of PLTs and of the homeodomain transcription factor WUSCHEL‐RELATED HOMEOBOX 5 (WOX5) on QC quiescence is linked to ROS. The identity of the stem cell niche in Arabidopsis is maintained by the spatial restriction of PROHIBITIN 3 (PHB3). Although WOX5 expression is confined to the QC, the WOX5 protein can move to the adjacent CSC layer to preserve the undifferentiated status of these distal stem cells.

The expression of PHB3 is regulated by the ERF109, ERF114 and ERF115 transcription factors (Kong et al., 2018). ERF115 is regulated by ROS as well as brassinosteroid signalling, whereas ERF109 is important in the transmission and amplification of ROS signals. PHB3 suppresses ERFs and maintains the activity of PLT1 and PLT2 via ROS to control root stem cell niche identity.

The SHORT‐ROOT (SHR) transcription factor, which is essential for the correct positioning of the QC, increases ROS production either by the activation of SA accumulation or by the direct activation of RBOHD, RBOHE and RBOHF (Li et al., 2020). SHR functions in parallel with PLT/WOX5 to enable ROS‐mediated SCN regulation and QC quiescence via the activation of RBOHD and RBOHF expression. Salicylic acid (SA)‐induced ROS production by RBOHs leads to supernumerary QC divisions through the repression of PLT1, PLT2 and WOX5 (Wang et al., 2021).

The regulation of ROS levels directly influences the ability of SHR to induce the periclinal divisions necessary for the formation of the cortex and endodermis. SHR maintains ROS levels in the RAM in order to create the micro‐environment required to mediate asymmetric periclinal cell divisions. RBOHD and RBOHF functions have also been linked to the SA‐mediated downregulation of PLTs and WOX5 in order to induce cell divisions in the QC (Wang et al., 2021).

Little is known about the organization and control of micro‐environments but it will be interesting to explore the role of biomolecular condensates that concatenate macromolecules locally and allow the spatiotemporal organization of biochemical reactions and cell signalling. This process often involves the intrinsically disordered regions of transcription factors and signal transduction proteins such as the plant‐specific NAC (NAM, ATAF1/2 and CUC2) transcription factors involved in seed germination and seedling establishment. For example, the regulated production of hydrogen peroxide in the shoot apical meristem triggers reversible protein phase separation in the TERMINATING FLOWER (TMF) transcription factor, which represses the expression of the floral identity gene *ANANTHA* to regulate flowering transition, thus maintaining the meristem in a vegetative state (Huang et al., 2021). This regulation involves highly conserved cysteine residues within TMF, which form disulfide bonds that concatenate multiple TMF molecules and elevate the number of intrinsically disordered regions to drive phase separation. This enables TMF to bind to the promoter of a floral identity gene *ANANTHA* and so repress its expression. This reversible transcriptional condensation mechanism provides added flexibility for gene control in response to developmental cues. The redox‐regulated formation of molecular condensates that control gene expression and other key processes may explain why quiescent cells are largely oxidized in plant meristems, even when oxygen is limiting.

## SMALL SIGNALLING PEPTIDES – A ROLE FOR CEP/CEPR/CEPD IN SYSTEMIC, NUTRIENT‐LIMITED QUIESCENCE

Mechanisms by which nutrient limitation regulates cell division in higher plants are beginning to emerge, including the role of small signalling peptides (reviewed by Jeon et al., 2021; Jourquin et al., 2020). The peptides CLAVATA 3 (CLV3)/EMBRYO‐SURROUNDING REGION‐RELATED (CLE) and ROOT MERISTEM GROWTH FACTOR (RGF), for example, have well‐defined functions in the non‐cell‐autonomous behaviour of meristem activity in response to abiotic cues, particularly hormones. More recently, the C‐TERMINALLY ENCODED PEPTIDE (CEP) family of polypeptides have emerged as integrators of nutrient and sugar status (Jeon et al., 2021; Jourquin et al., 2020). Low soil nitrogen or changing sucrose availability triggers the expression of *CEP* family genes in the roots of Arabidopsis (Delay et al., 2013) and *Medicago* (Imin et al., 2013). The roots of Arabidopsis *cep3* plants show increased primary root length and lateral root density in nitrogen‐deficient conditions, whereas ectopic CEP3 peptide attenuates growth (Delay et al., 2013, 2019). CEP3 depletes the number of dividing cells in the RAM, apparently resulting in G1 arrest (Delay et al., 2019). The proportion of S‐phase cells in the RAM of *CEP3oe* plants is highly dependent on the availability of nitrogen, whereas *cep3‐1* plants are relatively insensitive to nitrogen depletion, showing a sustained number of S‐phase cells (Delay et al., 2019).

The mechanism of this function requires root–shoot reciprocal communication. In conditions of low nitrogen, the CEP protein translocates via xylem to the shoot, interacting with CEP RECEPTOR 1 (CEPR1) leucine‐rich repeat receptor kinases (LRR‐RKs; Tabata et al., 2014) in the vascular tissues of leaves. In turn, this signal triggers the expression of CEP DOWNSTREAM 1 (*CEPD1*) and *CEPD2* glutaredoxins, which are transported via the phloem back to the roots (Ohkubo et al., 2017). In root apical and lateral meristems, where nitrogen is available, CEPD activates the expression of nitrate transporters (*NRT2.1*) to systemically compensate for areas of low nitrogen. In areas of low nitrogen, it appears that CEPD represses *NRT2.1* and cell division, leading to quiescence.

High levels of sucrose induce *CEP* expression and repress the growth of apical and lateral root meristems. Hence, CEP may be considered to transduce the perception of changing C:N ratios. CEP3 causes rapid G1‐arrest in RAM cells in carbon‐depleted conditions and attenuates the resumption of the cell cycle following the addition of glucose (Delay et al., 2019). This was shown to be dependent upon metabolizable sugars only (Chapman et al., 2019). The influences of CEP3 on sugar signalling is independent of the TOR kinase because the phosphorylation of S6K1 remained responsive to glucose resupply in carbon‐starved roots (Delay et al., 2019). It is conceivable then that the CEP/CEPR signal influences quiescence via the SnRK1 module, as supported by transcriptional data (Chapman et al., 2019). However, a further understanding of the relationship between nitrogen and sugar availability and their metabolism with quiescence is required. For example, Liu et al. (2021) recently showed that TOR kinase in the shoot apex is responsive to nitrogen availability. The role of CEP/CEPR in the latter mechanism is unknown, but the differential regulation of the TOR kinase in root and shoots has been demonstrated in different hormone‐ and light‐signalling contexts (Li et al., 2017).

Taken together, the CEP/CEPR/CEPD module has emerged as a mechanism for signalling changes in nutrient and energy to effect local quiescence and the redirection of sink strength in roots (Figure 1). ROS signals produced by mitochondria regulate cell cycle progression and gene expression in the nucleus (Figure 1). In addition, ROS accumulation inhibits SnRK1 kinase activity, leading to the termination of hypoxia‐induced SnRK1 functions. Moreover, the auxin‐dependent activation of the RAC/ROP (Rho‐GTPase) proteins leads to apoplastic ROS production through interactions with NADPH oxidases (Figure 1). ROP2 positively regulates TOR kinase in response to ROS and sugars (Figure 2). Although TOR kinase activation is promoted under low glucose conditions, an attenuation of TOR kinase activity, linked to metabolic ROS accumulation, can occur at high glucose levels. Together with NO and nitrate reductase activity, ROS signals regulate homeostasis in the root stem cell niche (Landrein et al., 2018; Wany et al., 2018). Low nitrogen availability causes root quiescence, whereas nitrogen acquisition from areas of sufficiency is activated (Ohkubo et al., 2017; Tabata et al., 2014). In the case of sucrose, the CEP/CEPR/CEPD module appears to repress the response to elevated energy levels in roots. Most recent insights show that under nitrogen‐deplete conditions, the CEPD integrates signals from both CEP/CEPR and cytokinin (Ohkubo et al., 2017; Taleski et al., 2022). However, whether the influence of elevated sucrose operates via the same mechanism is not clear and, further, the mechanism by which CEPD influences cell division remains largely elusive (Taleski et al., 2022). CEPD1 and CEPD2 have been identified by homology as class‐III glutaredoxins; however, whether they function as glutaredoxins remains to be demonstrated.

## CONCLUSIONS AND PERSPECTIVES

**Figure 3:**
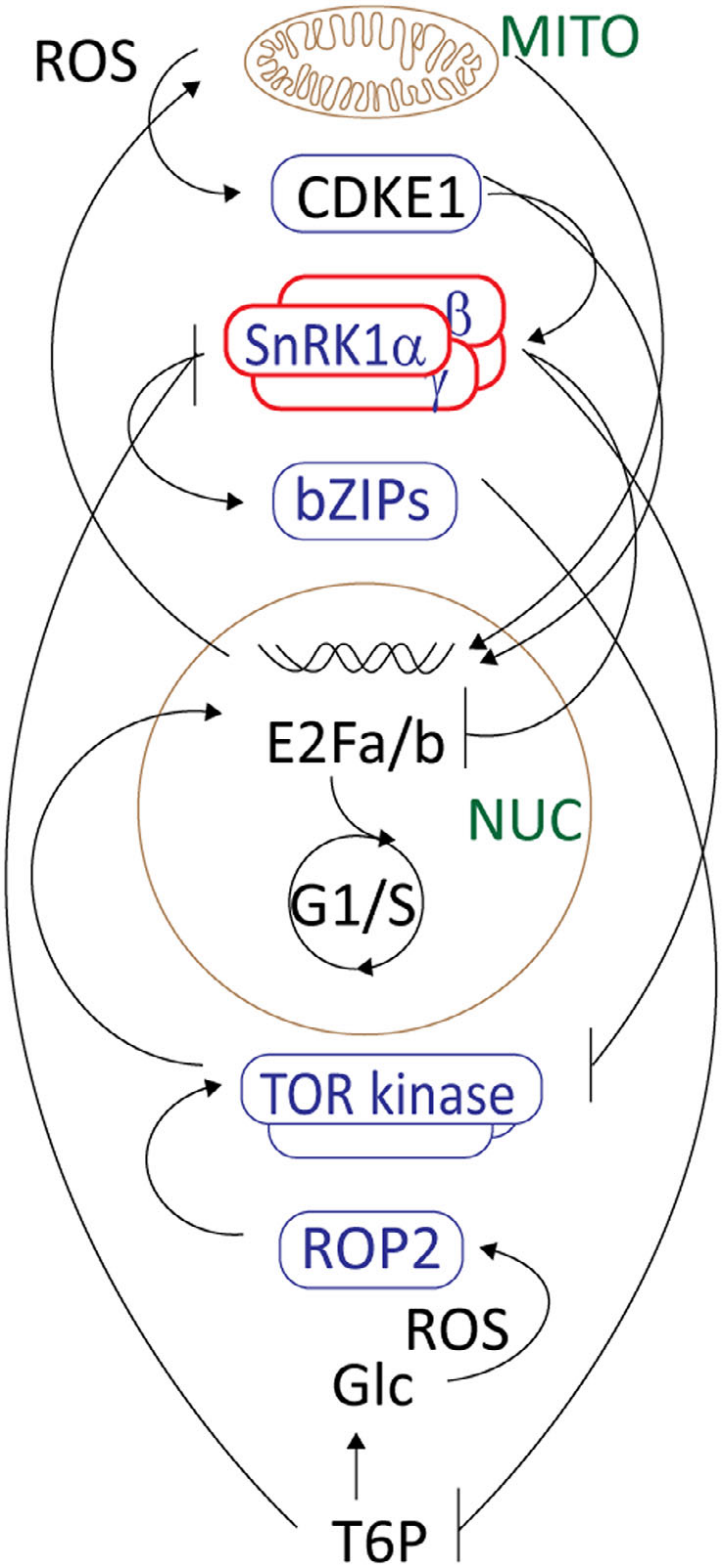
A simplified overview of the integration of metabolic regulation with the regulation of cell division in meristems. SnRK1 plays a central role and potentially represents the default mode in restraining cell division by several mechanisms, including the repression of cell cycle activation (E2Fa/b) as well as TOR kinase, and mechanisms dependent on the SnRK1 activation of bZIP transcriptional regulation, which in turn represses the availability of inhibitors of SnRK1, including sugar phosphates (T6P). SnRK1 also plays a role in transducing the retrograde regulation of gene expression and quiescence of metabolism and cell division by mitochondria (CDKE). ROS signalling plays important roles in pro‐ and anti‐proliferative signals. Abbreviations: CDKE1, cyclin‐dependent kinase E1; MITO, mitochondria; NUC, nucleus.

The above discussion has highlighted how our knowledge of the metabolites, proteins and genes that regulate, maintain and release quiescence has increased in recent years, particularly those related to ROS/redox and hypoxia signalling. Similarly, we now have a much clearer picture of how metabolic constraints on mitochondrial metabolism result in changes in nuclear gene expression that regulate the TCA cycle, respiratory electron transport system and associated processes. Quiescence is entrained in meristematic tissues by a combination of the regulation of energy and oxygen‐dependent metabolism/signalling, as illustrated in Figure 3. Apoplast/cell wall processes and the regulation of plasmodesmal aperture size may play a key role in this regulation by limiting oxygen permeability and the movement of signalling molecules through intracellular pathways; however, this remains poorly characterized and poorly understood. Within the meristem, stem cells reside in a low‐oxygen but relatively oxidized environment without any loss of viability or function. In contrast, when these changes are imposed by stress‐induced quiescence, cells are less able to withstand low oxygen levels and high ROS conditions, leading to autophagy and programmed cell death (Box 3).

Box 3 **Summary**

Quiescence in higher plants is the reversible repression of meristem activity. This process influences cell‐to‐cell communication on local and distal tissues. SnRK1 plays a central role in the metabolic regulation of quiescence, as it functions in different subcellular locations to integrate cues directly from metabolites, particularly T6P, as well as mitochondrial signals, and represses the activity of the E2Fa/b replication licencing transcription factor as well as the TOR kinase. ROS and hypoxic signalling generated in the apoplast and mitochondria provide context specificity, together with sugar and peptide signals, whereas the mechanistic function of plasmodesmata in regulating metabolic control remains to be explored. Growth‐promoting cues re‐establish cell division by altering the homeostasis of ROS and sugar signals, repressing SnRK1 and activating the TOR kinase.

Quiescence is controlled by precise on/off switches of gene repression and activation, which requires the integration of endogenous and environmental signals into fine‐tuned spatial/temporal gene expression in a stable environment. Although significant progress has been made in the field in recent years, the gaps in our knowledge are evident, particularly regarding the overall hierarchy of events, the spatial and temporal regulation of signalling and, crucially, how these processes function in a concerted fashion to determine cell fate.

## CONFLICT OF INTEREST

The authors declare that they have no conflicts of interest associated with this work.
